# Production of a natural color through microwave‐assisted extraction of saffron tepal's anthocyanins

**DOI:** 10.1002/fsn3.978

**Published:** 2019-03-18

**Authors:** Seid Mahdi Jafari, Katayoun Mahdavee Khazaei, Elham Assadpour

**Affiliations:** ^1^ Department of Food Materials and Process Design Engineering Gorgan University of Agricultural Sciences and Natural Resources Gorgan Iran

**Keywords:** anthocyanins, microwave‐assisted extraction, natural color, saffron

## Abstract

The extraction of anthocyanins from saffron (*Crocus sativus*) flower's tepal by microwave‐assisted extraction (MAE) was studied. The independent factors were solvent to sample ratio (10:1‒100:1), extraction temperature (35‒75°C), and time (5‒15 min). Maximum irradiation power in all experiments was 360 W. We applied response surface methodology (RSM) in order to determine optimum processing conditions which give maximum extraction efficiency (mg cyanidin‐3‐glucoside/g dried tepals). It was found that the influence of solvent ratio was more important for extraction yield than two other variables. Extraction conditions which maximized the extracted anthocyanins content were ratio of solvent to sample 77.5 ml/g, temperature 48°C, and extraction time of 9.3 min that resulted in 101 mg anthocyanins/g. In addition, MAE was a rapid and efficient technique for saffron anthocyanins due to disruption of cell walls under microwave irradiation, which was observed by microstructural analysis.

## 1 INTRODUCTION

Nowadays, there are more tendencies to apply natural colorants instead of synthetic ones in the food and other industries (Akhavan, Jafari, Ghorbani, & Assadpoor, 2014). Functional properties and attractive color of anthocyanins make them suitable substitutes for synthetic anthocyanins in the food industry (Akhavan Mahdavi, Jafari, Assadpoor, & Dehnad, 2016; Akhavan Mahdavi, Jafari, Assadpour, & Ghorbani, 2016). Anthocyanins after isolation from their natural resources are very vulnerable, and stability of their chemical structure can be influenced by different factors such as time, concentration, pH, storage temperature, oxygen, light, presence of enzymes, solvents, flavonoids, proteins, and metal ions (Giusti & Wrolstad, 2001; Khazaei, Jafari, Ghorbani, Kakhki, & Sarfarazi, 2016). Therefore, there is a need to a technique to extract anthocyanins fast, affordable, easy, and by the maximum amount.

Anthocyanin extraction through traditional solvent extraction which has been used widely is time consuming, with a low efficiency, and requires a high solvent consumption. In addition, extraction overheating for a long time during solvent extraction can cause degradation of anthocyanins (Lapornik, Prošek, & Wondra, 2005). Modern techniques of extraction including ultrasound‐assisted extraction (UAE) and microwave‐assisted extraction (MAE) as compared with traditional extraction have had higher performance especially in combination of solids and high polarity environments such as phenolic compounds.

Microwave‐assisted extraction requires less solvent and less time for extraction. MAE works based on two mechanisms for energy transfer namely dipole rotation and ionic conduction (Routray & Orsat, 2014). The electromagnetic radiation of microwave energy results in destruction of cell wall matrix and quickly increases the solvent penetration into the plant cells which leads to the leaching of ingredients during the microwave heating process (Maran et al., 2015). Different chemical ingredients absorb microwaves in a different extent, and this behavior makes MAE an efficient method for extractions; more importantly, it makes it feasible to selectively extract target bioactive compounds from the complex food matrices (Setyaningsih, Saputro, Palma, & Barroso, 2015). During MAE, heat transfer occurs from the material to the bulk solvent in MAE and is distributed volumetrically throughout the irradiated sample. In this phenomenon, heat is created inside the microwave‐absorbing substances and is transferred to the extracting medium. The concurrence of the two transport phenomena, namely heat and mass transfer, makes the extraction rate of MAE many orders of magnitude higher than that of conventional solvent extraction and results in much shorter extraction times (Bakhshabadi et al., 2017; Rafiee, Jafari, Alami, & Khomeiri, ; Taghvaei, Jafari, Assadpoor, Nowrouzieh, & Alishah, 2014). Factors like type and volume of the solvent, solvent to sample ratio, sample moisture, extraction time, exposure time, size of particles, soaking samples in solvent before extraction, and dielectric constant of the solvent affect the MAE efficiency. Soaking the samples before MAE makes solvent to penetrate into the tissue of sample (Sun, Liao, Wang, Hu, & Chen, 2007; Xu & Howard, 2012).

Saffron, the red stigmas of *Crocus sativus* L., is the most expensive spice in the world (Sarfarazi, Jafari, & Rajabzadeh, 2015) and is produced largely in Iran so that its annual production is more than 90% of total saffron in the world (Rajabi, Ghorbani, Jafari, Mahoonak, & Rajabzadeh, 2015; Shahi, Assadpour, & Jafari, 2016). It has cyanic color flowers with major colorant of anthocyanins (Norbaek, Brandt, Nielsen, Orgaard, & Jacobsen, 2002). Since approximately 86.4% (wet base) or 96.36% (dry base) of total weight of saffron flowers belong to their tepals, then in saffron season after picking stigmas, a large scale of this huge anthocyanin resource is discarded into the nature as a waste. Extracted anthocyanins of saffron tepals can be used in confectionary as natural dyes, also in pharmacy or other industrial applications (Kafi, 2006). A major drawback of natural anthocyanins is their instability against changes in pH, light, oxygen, humidity, etc., and some strategies have been applied by the researchers to increase their stability such as co‐pigmentation (Babaloo & Jamei, 2017; Tan, Celli, & Abbaspourrad, 2018; Tan, Selig, & Abbaspourrad, 2018) and encapsulation (Jafari, Mahdavi‐Khazaei, & Hemmati‐Kakhki, 2016; Khazaei, Jafari, Ghorbani, & Hemmati Kakhki, 2014).

Response surface methodology (RSM) is as a statistical technique to build approximated models based on data collected during physical examination, simulating by software and experimented observations. In RSM, a group of mathematical‐statistical models are applied for engineering and modeling procedures. The main goal of this methodology is to optimize response surface which is influenced by the process factors (Yolmeh & Jafari, 2017).

The specific aims of this research were to develop a microwave‐assisted process for extraction of anthocyanins from saffron tepal and to investigate and optimize the effect of process variables (temperature, time, and solvent ratio) and their interactions on the response (anthocyanin yield), by using RSM so that maximum amount of anthocyanins can be extracted and presented as a natural color for the food and pharmaceutical industries.

## 2 MATERIALS AND METHODS

Saffron flowers were gathered from a farm near Torbat‐E‐Heydariyeh (Khorasan‐Razavi, Iran). After separating stigmas and anther, the tepals were dried in a dark and warm room (32ºC ± 2) exposed to a fan. Dried tepals were grinded and sieved (16 meshes) and packaged in air‐tight bags in 5ºC. Analytical grade hydrochloric acid (PubChem CID: 28153) and ethanol (ethylene glycol, PubChem CID: 174) were purchased from Merck (Darmstadt, Germany). For preparation of all solutions, distilled water was used. Potassium chloride (PubChem CID: 4873) buffer pH 1.0 and sodium acetate (PubChem CID: 517045) buffer pH 4.5 were of analytical grade.

### 2.1 MAE of anthocyanins

Microwave‐assisted extraction procedure used in the experiments was adopted from Sun et al. (2007) with some modifications. In all extraction experiments, dried powder of saffron tepal was applied based on the designed ratios (Table 1) into 30 ml acidic ethanol (50% or 25% ethanol acidified with HCL 0.1 N up to pH = 2) (g/ml) as the solvent in flasks and soaked for 15 min in ambient temperature before processing. During MAE, closed flasks were heated in a microwave oven (Microsynth‐Milestone, USA) with different temperatures and time periods based on design experiments (Table 1) plus 10 min before processing to set extraction condition and 10 min after extraction to return pressure of the microwave oven to the ambient pressure. Pressure and power of irradiation within the microwave oven were controlled automatically to achieve goal temperatures; maximum power was 360 W in all experiments.

**Table 1:** Observed response values with different combination of extraction time (*X*_1_), temperature (*X*_2_), and ratio of solvent to dried saffron tepal (*X*_3_) used in central composite rotatable design (CCRD) Trough MAE

Run no	Block	Factor values			Response values^a^ (mg/g petal)
		*X*_1 _(min)^b^	*X*_2 _(°C)	*X*_3 _(ml/g)	
1	1	12.5 (1)	45 (−1)	77.5 (1)	97.05
2	1	7.5 (−1)	45 (−1)	32.5 (−1)	41.67
3	1	12.5 (1)	45 (−1)	32.5 (−1)	41.58
4	1	7.5 (−1)	65 (1)	32.5 (−1)	37.66
5	1	12.5 (1)	65 (1)	32.5 (−1)	39.51
6	1	10 (0)	55 (0)	55 (0)	71.81
7	1	10 (0)	55 (0)	55 (0)	71.32
8	1	7.5 (−1)	45 (−1)	77.5 (1)	97.07
9	1	12.5 (1)	65 (1)	77.5 (1)	95.29
10	1	7.5 (−1)	45 (−1)	77.5 (1)	101.52
11	1	7.5 (−1)	65 (1)	77.5 (1)	91.04
12	2	10 (0)	55 (0)	55 (0)	71.91
13	2	10 (0)	55 (0)	55 (0)	71.73
14	2	15 (2)	55 (0)	55 (0)	66.41
15	2	10 (0)	75 (2)	55 (0)	54.80
16	2	10 (0)	35 (−2)	55 (0)	67.35
17	2	10 (0)	55 (0)	100 (2)	130.81
18	2	5 (−2)	55 (0)	55 (0)	70.57
19	2	10 (0)^b^	55 (0)	10 (−2)	68.96

a Data expressed are the mean of triplicate analysis, and the unit of data in the last column is milligram cyanidin‐3‐glucoside equivalent in 100 ml extract.

b Numbers in parenthesis are coded symbol levels of independent parameters.

After extraction and cooling, samples were centrifuged (4,000 rpm, 10 min), filtered (filter paper Whatman No.1) and the total anthocyanins of supernatant were measured. The influence of different independent variables on total anthocyanin content of the extract was evaluated by a quadratic regression with Equation (1).

(1) $$Y=b_{0}+b_{1}x_{1}+b_{2}x_{2}+b_{3}x_{3}+b_{11}x_{1}^{2}+b_{22}x_{2}^{2}+b_{33}x_{3}^{2}+b_{1}b_{2}x_{1}x_{2}+b_{1}b_{3}x_{1}x_{3}+b_{2}b_{3}x_{2}x_{3}+\varepsilon$$

### 2.2 Determination of total anthocyanin content of extracts

Total anthocyanin content (TAC) of tepal extracts was analyzed with pH differential method that was adopted from Giusti and Wrolstad (2001). Briefly, the extracts were added into buffer solutions of pH = 1.0 and 4.5 and allowed to equilibrate for 20 min. Then, the spectrophotometric absorbance of each equilibrated solution was determined at 520 nm (λ max) and 700 nm for haze correction, through an UV–Vis spectrophotometer (Shimatzu‐160A–Japan). Anthocyanin content in acidic ethanol was measured based on cyanidin‐3‐glucoside (Lee, Durst, & Wrolstad, 2005). The absorbance of the diluted sample (*A*) was determined according to Equation (2):

(2) $$A={\left(A_{\text{vis}-\text{max}}-A_{700}\right)}_{\text{pH}\;1.0}-{\left(A_{\text{vis}-\text{max}}-A_{700}\right)}_{\text{pH}\;4.5}$$

TAC in the original sample was determined using Equation (3):

(3) $$\text{TAC}\left(\text{mg/L}\right)=\left(A\;\times \text{MW}\times \text{DF}\times 1,000\right)/\left(\varepsilon \times 1\right)$$

where MW is the molecular weight of Cyanidin 3‐glucoside*,* DF is dilution factor, and ɛ is molar absorptive of delphinidin.

### 2.3 Analysis of total anthocyanin content in saffron's tepal

5 ± 0.01g powdered samples of tepals were mixed with 200 ml HCl‐50% ethanol (pH = 2). The temperature during extraction was set at 25 ± 1ºC. This process was carried out for 8 hr while it was mixing slowly (100 rpm). Final extract was purified with a Whatman No.1 filter paper under vacuum and poured into a volumetric flask. The residue was reused and extracted again with the same procedure. Anthocyanin content of all obtained extracts was mixed and used for measurement of the total anthocyanins.

### 2.4 Statistical methods

For estimation of the effect of independent variables on extracted anthocyanins, response surface methodology (RSM) was applied through Design‐Expert version 6.0.2 (Statease Inc., Minneapolis, USA). Central composite rotatable design was suggested consisting of two blocks (solvent of ethanol 50% in treatments of block 1 and ethanol 25% in treatments of block 2) and 19 experiments as shown in Table 2 with four replicates in center point (Koocheki, Taherian, Razavi, & Bostan, 2009). A polynomial model was fitted to the data (Equation (3)) where *b_n_* was constant regression coefficient; *y* was the response (mg cyanidin‐3‐glucoside/g tepal); and *X*_1_, *X*_2_, and *X*_3_ were independent variables titles time, temperature, and solvent ratio, respectively. Statistical significance of each term in the regression equations was determined. Surface plots and counter plots were depicted with the same software. SPSS software was used to determine significant differences between data on center points in two different blocks.

**Table 2:** Regression coefficients for TAC of saffron tepal extract in quadratic model with MAE

Term	Coefficient	Standard error for the coefficient	*F* value	*p* value
Intercept	72.64	11.76		
Block 1	−2.44			
Block 2	2.44			
*X*_1_‐time	−0.42	1.68	0.664	0.8080
*X*_2_‐temperature	−2.70	1.68	2.60	0.15
*X*_3_‐solvent ratio	23.89	1.68	203.07	<0.0001
$x_{1}^{2}$	−1.78	1.51	1.39	0.2768
$x_{2}^{2}$	−3.65	1.51	5.83	0.0465
$x_{3}^{2}$	3.94	1.51	6.8	0.0350
*X*_1_ *X*_2_	1.33	2.37	0.31	0.5923
*X*_1_ *X*_3_	−0.24	2.37	0.011	0.9206
*X*_2_ *X*_3_	−0.77	2.37	0.11	0.7541

## 3 RESULTS AND DISCUSSION

### 3.1 Total anthocyanin content of saffron tepals

Total anthocyanin content in the extract was equal to 136.96 ± 5.7 mg cyanidin‐3‐glucoside/g tepal. Also Khazaei et al. (2014) showed that saffron tepals were composed of 8.7% protein, 10.25% lipid, 11.39% fiber, 13.05% total sugar, and 5.76% total ash with 87.11% moisture content.

### 3.2 Modeling of anthocyanin extraction by MAE

Obtained TAC through MAE experiments is represented in Table 1. Statistical analysis on four replicates in central point (10 min, 55°C, and 55 ml solvent/g) did not show any significant differences (*p* > 0.01). It reveals that two different solutions of acidic ethanol (50% and 25%) did not have significant effects (*p* > 0.01) on anthocyanin extraction from saffron tepals through MAE. After obtaining ANOVA and based on *F* and *p* values, a second‐order polynomial response surface model (Equation (3) and Table 2) was fitted to the response variable (*Y*). Based on ANOVA test (Table 2), solvent ratio had a significant (*p* < 0.01) linear (*X*_3_) and quadratic ($x_{3}^{2}$) (*p* < 0.05) effect on the model. Also quadratic effect of temperature ($x_{2}^{2}$) with 95% confidence was significant. Represented model (Equation (4)) was a second‐order polynomial equation (regression model) after removing insignificant coefficients in 95% confidence level. The value of 72.64 was the constant coefficient of model.

(4) $$Y=72.64+23.89x_{3}-3.65x_{2}^{2}+3.94x_{3}^{2}\;\left(5\%\;\text{level}\right)$$

Polynomial coefficients of the independent variables regardless of their positive or negative changes in the response indicate their importance on TAC. As it is obvious, among three independent variables, solvent ratio derived significant and positive linear and quadratic effects on TAC and temperature showed a negative quadratic effect on MAE condition. Coefficient of determination (*R* ^2^) was 0.97 (near to unit), and adjusted *R* ^2^ was 0.93 showed that fitted empirical model was appropriate for real data and the model can describe the relation among variables (Koocheki et al., 2009).

### 3.3 Response surface plots of MAE

**Figure 1:**
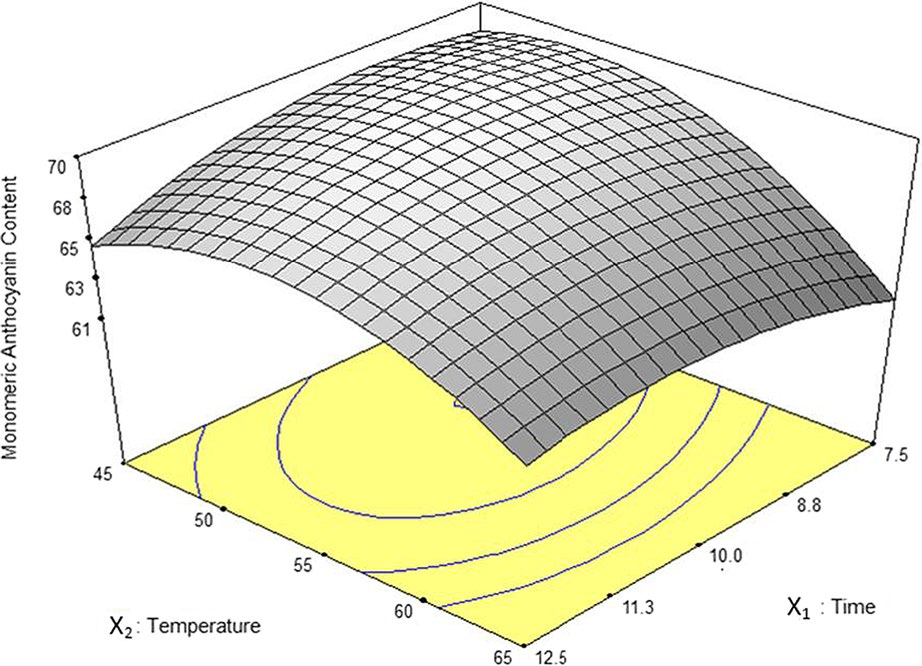
Response surface plot for anthocyanin yield as a function of extraction time and temperature in solvent ratio to sample 55 ml/g through MAE

The influence of temperature and exposure time on extraction yield is shown in Figure 1. Total anthocyanins mounted up with rising temperature (irradiated power which was controlled automatically by apparatus) in a cured shape and achieved a maximum value; then, it was decreased. For all extraction times, higher temperature more than 50°C resulted in a reduction in TAC. Anthocyanins at higher temperatures could have been deteriorated and polymerized to get brown or colorless. Due to stability of polymeric anthocyanins in various pH values, they cannot be determined by pH differential method, and consequently, total measured anthocyanins are reduced (Giusti & Wrolstad, 2001). Generally, at most temperatures, loss of TAC is happening with elevating extraction time higher than 10 min. This may be explained by the dominance of damaging effects of temperature in comparison with time on TAC in intervals. In the results revealed by Sun et al. (2007), they approved that extraction temperature or irradiated power plays a major role in TAC of extracts through MAE.

**Figure 2:**
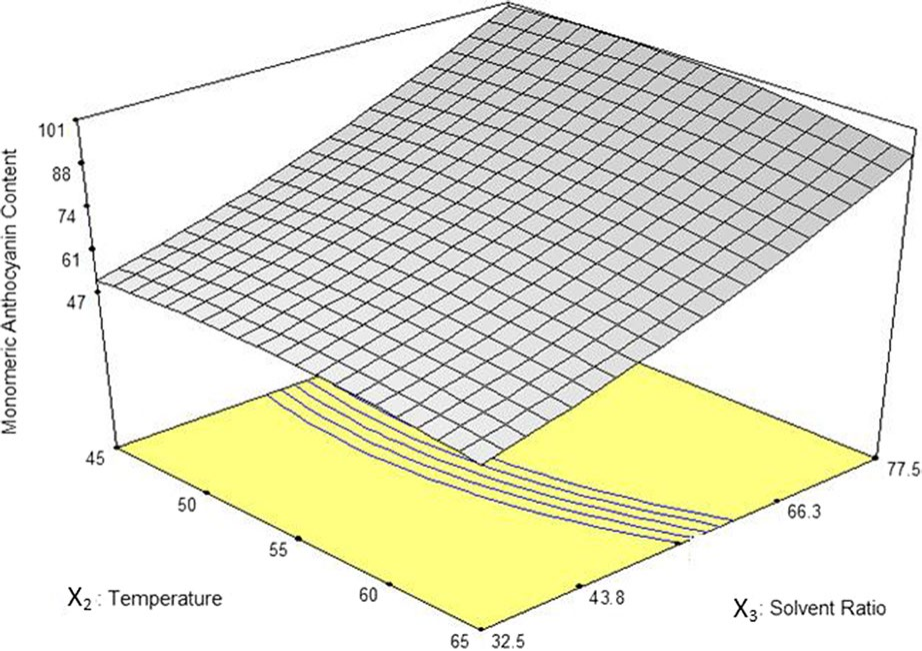
Response surface plot for anthocyanin yield (T_Acys_) as a function of extraction temperature and solvent ratio in 10 min through MAE

Interactive effect of temperature and solvent ratio on TAC has shown in Figure 2. The maximum anthocyanin content was extracted in the highest solvent ratio (77.5 ml/g). Higher solvent ratio results in a higher density gradient and higher distribution coefficient, then, causing a faster release of tepal tissue's extract and, hence, higher TAC. Moreover, higher density of anthocyanins within the extract at smaller solvent ratios results in more reactions between monomeric anthocyanins and other chemical compounds of tepals tissue; thus, stability of anthocyanins declines and resulting lower TAC (Lee et al., 2005). Increasing temperature more than 45°C in every solvent ratio did not show a meaningful effect on TAC of the extract. In a research by Ma et al. (2009) for extraction of flavonoids (anthocyanins are coloring materials belong to a big chemical group named flavonoids) from Radix astragali by MAE, increased power from 200 to 1,000 W caused excessive heat production and destructed flavonoids and reduced extraction yield.

**Figure 3:**
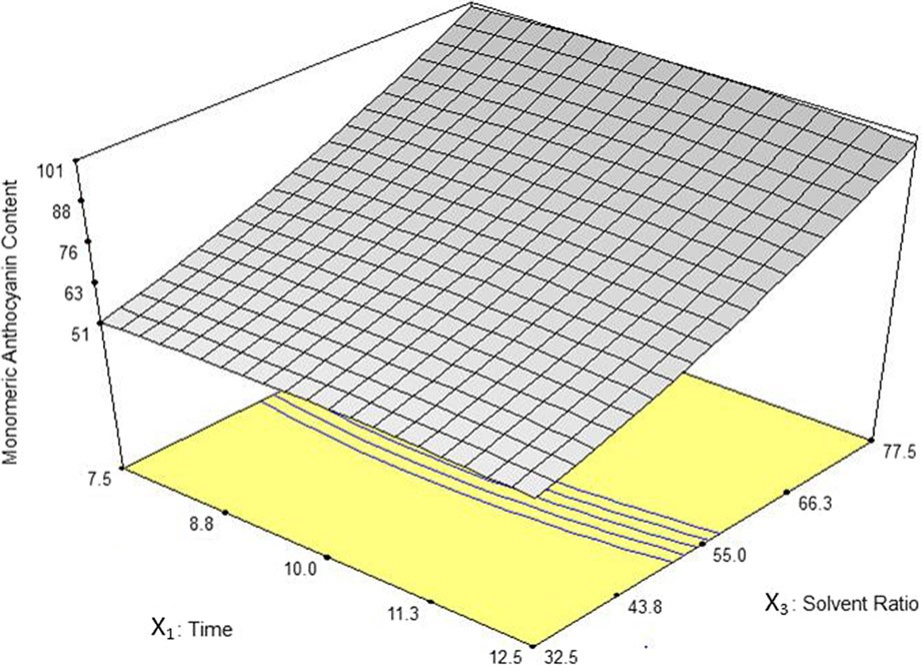
Response surface plot for anthocyanin yield as a function of extraction time and solvent ratio in 55°C through MAE

The influence of extraction time and solvent ratio on TAC has been illustrated in Figure 3. It was found that the highest extraction efficiency was related to maximum solvent ratio (77.5 ml/g). In general, the highest TAC through different solvent ratios was determined to be around 10 min of extraction. Cacace and Mazza (2003a) observed that at higher solvent to solute ratios, the amount of extracted phenolic compounds from black currant was increased and the extraction time decreased. They also reported similar results in extraction of phenolic compounds from milled berries (Cacace & Mazza, 2003b).

### 3.4 Optimization of anthocyanins extraction

For achieving the highest amount of TAC during extraction, optimal conditions of the MAE process were determined. The lower and upper limits of anthocyanins were equal to 52 and 102 mg/g, respectively. Solution provided by Design‐Expert considering highest desirability also highest cost effective extraction condition in cases of the same desirability index (usage of shorter extraction time, lower temperature, and less solvent) was as follows: extraction time of 9.3 min, temperature of 48°C, and solvent ratio of 77.5 ml/g (with ethanol concentration of 50% or 25%) (Table 3); in these conditions, the value of extracted anthocyanin was 101.0 mg/g. In a previous study by Khazaei et al. (2016), optimum extraction conditions that resulted in maximized anthocyanins by conventional soaking extraction were found to be a ratio of solvents to sample 20 ml/g, ethanol concentration of 25.02%, temperature 25.8°C, and extraction time 24 hr which gave 160.9 mg/g.

**Table 3:** Predicted optimum conditions for the extraction of saffron tepal extract and responses through MAE method

Factors	Low	High	Optimum
Solvent ratio (ml/g)	32.5	77.5	77.5
Extraction temperature (ºC)	35	75	48
Time (min)	7.5	12.5	9.3
Total monomeric anthocyanin (mg/g)	52	102	101

To assess the compliance of optimal extraction conditions with laboratory results, extraction was performed in the optimum extraction conditions suggested by the software. Average of TAC from three replicates was equal to 105.32 ± 6.4 mg/g where it was 77% of total monomeric anthocyanin content of saffron tepals (136.96 ± 5.7 mg/g). According to standard deviation, obtained TAC in experimental conditions was almost the same as the results predicted by the model and revealed a good fitness to the predicted data.

### 3.5 Microstructural analysis of extracted tissues

**Figure 4:**
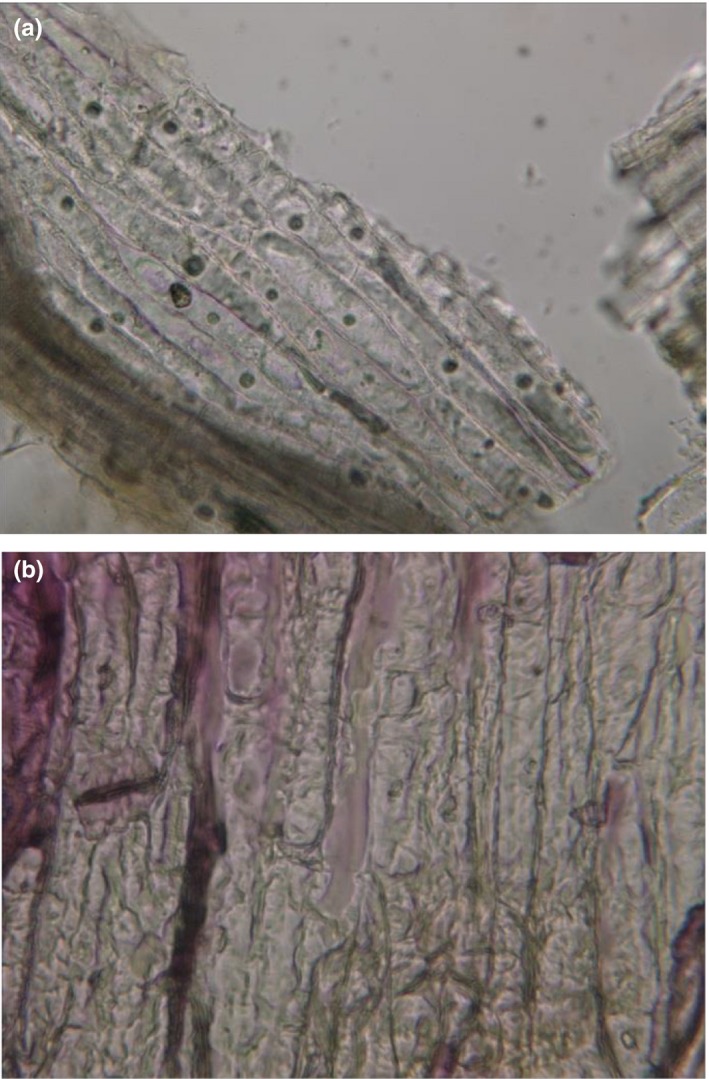
Microscopic images (400×) of saffron tepals after extraction by (a) conventional soaking (15 min, 55°C) and (b) microwave‐assisted extraction (15 min, 55°C)

Microscopic photos taken from saffron tepals (Figure 4) showed cell wall destruction through MAE resulting in higher and rapid extraction of anthocyanins compared to conventional soaking extraction. The cells of saffron tepals were interrupted after MAE so that nuclei of the cells under the microscope were not visible (as shown in Figure 4b). Destruction of cell walls through MAE has been investigated previously by Rastogi, Raghavarao, Niranjan, and Knorr (2002). It seems that microwave heating destroys weak hydrogen bonds established between dipole molecules (Xu & Howard, 2012), and physical disruption of MAE causes cell walls to be more permeable and therefore makes anthocyanin compounds to transfer directly into surrounding solvent more easier (Sun et al., 2007).

## 4 CONCLUSION

Our results revealed that extraction of natural colors such as anthocyanins through modern techniques including microwave‐assisted extraction is much more efficient and economic in terms of time and solvent usage. From the studied process variables of temperature, time, and solvent ratio, the last factor had a statistically significant linear and quadratic effect on anthocyanins extracted from saffron tepals. Rising solvent ratio increased concentration gradient of anthocyanins and further increased extraction efficiency. The content of extracted anthocyanins was not affected by ethanol percent with concentrations in the range of 50%–25%. Maximum total anthocyanin content (TAC) was obtained in 9.3 min extraction time, 48ºC, and 77.5 ml solvent/g tepals. Extracted anthocyanins were 101.0 mg/g which was equal to 77% of total monomeric anthocyanin content. Microscopic photos taken from saffron tepal's showed cell wall destruction through MAE resulting in higher and rapid extraction of anthocyanins. To conclude, saffron tepals are very rich in anthocyanins and can be used as a potential natural color resource of anthocyanins for food and pharmaceutical industries.

## CONFLICT OF INTEREST

All authors declare that there is no conflict of interest.

## ETHICAL STATEMENT

There was no human or animal testing in this study.
